# Loading and Skeletal Development and Maintenance

**DOI:** 10.4061/2011/786752

**Published:** 2010-12-20

**Authors:** P. Bergmann, J. J. Body, S. Boonen, Y. Boutsen, J. P. Devogelaer, S. Goemaere, J. Kaufman, J. Y. Reginster, S. Rozenberg

**Affiliations:** ^1^Department of Nuclear Medicine, Laboratory of Clinical Chemistry and Experimental Medicine, CHU Brugmann, Université Libre de Bruxelles, 4 Pl. Van Gehuchten, 1020 Brussels, Belgium; ^2^Department of Medicine, CHU Brugmann, Université Libre de Bruxelles, 1020 Brussels, Belgium; ^3^Division of Gerontology and Geriatrics, Center for Musculoskeletal Research, Department of Experimental Medicine, Catholic Leuven University, 3000 Leuven, Belgium; ^4^Department of Rheumatology, Mont-Godinne University Hospital, Université Catholique de Louvain, 1200 Brussels, Belgium; ^5^Rheumatology Unit, Saint-Luc University Hospital, Université Catholique de Louvain, 1200 Brussels, Belgium; ^6^Unit for Osteoporosis and Metabolic Bone Diseases, Ghent University Hospital, 9000 Ghent, Belgium; ^7^Department of Public Health Sciences, University of Liège, 4000 Liège, Belgium; ^8^Department of Gynaecology-Obstetrics, Free University of Brussels, 1090 Brussels, Belgium

## Abstract

Mechanical loading is a major regulator of bone mass and geometry. The osteocytes network is considered the main sensor of loads, through the shear stress generated by strain induced fluid flow in the lacuno-canalicular system. Intracellular transduction implies several kinases and phosphorylation of the estrogen receptor. Several extra-cellular mediators, among which NO and prostaglandins are transducing the signal to the effector cells. Disuse results in osteocytes apoptosis and rapid imbalanced bone resorption, leading to severe osteoporosis. Exercising during growth increases peak bone mass, and could be beneficial with regards to osteoporosis later in life, but the gain could be lost if training is abandoned. Exercise programs in adults and seniors have barely significant effects on bone mass and geometry at least at short term. There are few data on a possible additive effect of exercise and drugs in osteoporosis treatment, but disuse could decrease drugs action. Exercise programs proposed for bone health are tedious and compliance is usually low. The most practical advice for patients is to walk a minimum of 30 to 60 minutes per day. Other exercises like swimming or cycling have less effect on bone, but could reduce fracture risk indirectly by maintaining muscle mass and force.

## 1. Introduction

The relations between mechanical usage and bone metabolism are well known since more than one century, when Wolff described in 1892 how bone modeling during growth was determined by local strains, to evolute towards the most adapted structure to resist mechanical stress [1]. Low gravity or disuse causes bone loss. Regional increase of solicitation leads to an increased bone mass. To take these observations into account, Frost proposed the concept of mechanostat [2, 3]: bone mass and geometry are regulated cybernetically by mechanical inputs. According to this concept, one condition to develop and maintain healthy bones is that the skeleton is submitted to mechanical strain and that sensing of these strains is efficient and correctly transmitted to competent effector cells, osteoclasts and osteoblasts. Mechanical loading and its sensing are important at all ages to build strong bones during childhood and adolescence, to maintain peak bone mass during early adulthood, and to minimize bone loss at the menopause and with ageing. In this paper, we shall summarize the present knowledge concerning the signals and effectors involved in the mechanoregulation of modeling and remodeling of bone. We shall examine how mechanical regulation is integrated with other regulators of bone metabolism: nutrition, hormones, and drugs; Eventually, we shall examine to which extent mechanical forces can help to improve bone mass at different ages of life.

This report is based on an extensive literature search through Pubmed and Medline, using as keywords mechanical loading, osteoporosis, bone formation, bone resorption, disuse osteoporosis, physical activity, sport. Several recent reviews were also consulted.

## 2. Experimental Models Demonstrating the Effects of Loading on Bone Remodeling

Different experimental models have been used to study in vivo how bone remodeling is affected by mechanical forces, for a large spectrum of mechanical deformation (<50–4000 *μ*strains). Loading of the sectioned bird ulna prevents the increase of cortical porosity and endosteal resorption induced by disuse, and induces new periosteal apposition which is maximal on the tension surface [4]. The effect of mechanical loading on periosteal bone formation is dependent of the strains generated [5] and is related to circumferential strain gradient [6]; it depends on the frequency (when the strain is lower, the frequency to obtain a significant effect must be higher) [7, 8]. Loading of the immobilized tibia by four point bending decreases bone loss caused by immobilization and increases periosteal formation [9]. When rat ulna is submitted to axial loading, resorption surfaces convert to periosteal apposition [10]. An increased periosteal formation has also been observed in a model of bended mice tibias, with a dependency on frequency for strain rates between 10 and 400 Hz [11]. Axial compression induces an increase of cancellous bone volume which is maximal in the proximal metaphysis of mice tibias [12] and in the distal metaphysis of the rabbit femur [13]. Axial compression also increases bone formation in the rat tail vertebrae loading model [14]. Low strain (<50 *μ*strain) at very high frequency (90 Hz), comparable to the signals generated by postural muscle dynamics [15], can increase trabecular bone formation rate in normal weight bearing rats, and restore it to normal in tail suspended rats, a model for weightlessness [16]. As with hormonal signaling, there is a desensitization to prolonged mechanical stimulation; sensitivity is restored if loading is intermittent [17, 18], and brief periods of loading are sufficient to enhance formation [19].

## 3. Sensing and Transducing

Many experimental data in vitro and in vivo point to the osteocytes network as the main sensor detecting strain in the bone tissue [20]. These cells, embedded in their lacunocanalicular system and largely distributed in bone, interconnected by gap junctions between their cell processes, connected with cells of the bone surface and of the bone marrow [21] are ideally placed to sense bone loading and to direct remodeling according to strains. Osteocytes are sensitive to biomechanical stress [22], particularly to fluid flow and shear stress induced by loading in the lacunocanalicular system [23, 24]. The sensitivity of osteocytes to shear stress is higher than that of osteoblasts [25]. Osteocytes die by apoptosis in the absence of loading [26] and their death is associated with local activation of resorption, because of the removal of inhibitory signals [27]. Conversely, when shear stress is sensed by osteocytes, it prevents their apoptosis [28] and induces signals which repress osteoclasts [29, 30] and increase osteoblast differentiation [31]. Mathematical models using finite elements analysis can describe cortical and trabecular remodeling, and the orientation of osteons, on the base of osteocytic control [32]. 

Different calcium channels [33] trigger intracellular signaling, through increased intracellular calcium and protein kinase C activation. Sensing is improved by cell-cell communication through gap junctions, which are increased by loading and by membrane structure and interaction of integrins with the cell cytoskeleton [20, 34]. The signals generated by shear stress are amplified by ATP secretion, acting on G-proteins linked purinergic receptors [35]. Intracellular transduction implies several kinases, with a particular role of the extracellular signal regulated kinase ERK, the inhibition of which hampers mechanical signaling [36]. Strain, as PTH and other stimulators of bone formation, also increases osteoblast *c-fos* transcription by interaction with several regions of the promoter [37]. Sensing of strain may interact with PTH signaling. For instance, Miyauchi et al. have documented a volume-sensitive Ca^2+^ influx in osteocytes, particularly along the osteocytes processes, which is potentiated by parathyroid hormone through adenylate cyclase activation [38]. Cooperation in sensing and in *c*-*fos *transcription may contribute to the synergistic effects of mechanical strains and PTH on bone metabolism [39–42]. 

Another key molecule for transducing mechanical loading in bone is the estrogen receptor *α* (ER_*α*_). In rat cortical bone, more than 90% of the osteocytes have been shown to express ER_*α*_; when the rat tibia is submitted to strain, ER_*α*_ is phosphorylated [43], probably through MAP kinase [44], and translocated to the nucleus and to the membrane [45]. The deletion of ER_*α*_ decreases the potential of osteoblast-like cells to respond to mechanical stimulation [46]. The effect of mechanical loading on explanted rat ulnae is enhanced by estrogens [47–49], as the response to loading of osteoblast-like cells from postmenopausal women [50]. 

As ER_*α*_ content is upregulated by estrogens, Lanyon suggested that decreased load sensing because of a decreased number of receptors could be a key factor in postmenopausal bone loss (resetting of the mechanostat) [51]. It is notable that both estrogen deficiency and the absence of mechanical loading induce osteocytes apoptosis and bone loss [26, 52, 53]. 

At the other end of the loading spectrum, excessive loading and damage increase osteocytes apoptosis, resulting in the initiation of damaged bone removal [54]. In vivo experiments in sheep have shown a significant interaction between the estradiol levels and strain on the cross-sectional properties at the midshaft of hind limb bones: cortical bone growth was 6 to 27% greater in exercised animals with elevated estradiol levels than in those with lower E2, and than in the sedentary group [55]. Contrarily, treatment of male rats with estrogens can decrease periosteal apposition induced by mechanical loading [56]. This could result from the fact that estrogens induce by themselves an increased mechanical resistance of bone, reducing the strain induced by mechanical solicitation [57]. ER_*α*_ polymorphism also seems to influence the effect of exercise on bone accrual in girls [58].

## 4. Transmission to Effector Cells (Figure 1)

Conditioned media from osteocytes can increase osteoblasts proliferation and differentiation [59]. Several chemical mediators have been identified, which can circulate in the lacunocanalicular network to reach the effector cells. Among putative mediators are nitric oxide (NO), prostaglandins E and I, sclerostin, IGF's, TGF*β*, RANK-L, and OPG.

First, shear stress induces the production of nitric oxide (NO) [60, 61]; nitric oxide synthase expression is increased in vivo in osteocytes after reloading in tail suspended rats [62]. NO suppresses osteoclasts activity [63] and promotes osteoblast activity [64]. The inhibition of nitric oxide synthase by L-NAME prevents the increase of periosteal formation induced by mechanical loading [65], suggesting a central role for NO in transduction. This idea is further supported by the fact that the reconstruction of bone after reloading tail suspended mice is impaired in mice knocked out for the inducible NO synthase [66]. 

Second, shear stress induces prostaglandin synthesis [67] by increasing the activity of the inducible cyclooxygenase (COX2), an increase which is dependent on the phosphorylation of the extracellular regulated kinase (ERK) [68, 69]. Prostaglandins stimulate osteoblastic activity through IGF's [70], and IGF's are increased early after a mechanical stimulation [71–73]. PGE and PGI inhibit directly osteoclasts activity [74], while they activate bone remodeling through cells of the osteoblast lineage [75]. 

Third, sclerostin (SOST), an osteocytic protein belonging to the TGF/BMP family which inhibits Wnt signaling, is decreased by mechanical stimulation [76]; as Wnt has an essential role in osteoblast proliferation and differentiation, the decrease of SOST could be a major signal to increase bone formation in response to loading [77, 78]. 

As far as resorption is concerned, besides a possible direct effect of prostaglandins E and I to inhibit osteoclast activity, the ratio of RANKL and OPG in osteocytes and marrow stem cells culture medium is decreased by strain [30, 79]. When mechanically stimulated, osteocytes also produce TGF_*β*_ which could also mediate osteoclastogenesis inhibition [80]. 

Besides the control of their activities by osteocytes, osteoblasts, and osteoclasts attach to bone matrix and the deformations of the bone surfaces (trabecular, endosteal, and periosteal) cause shortening and elongation of these cells. In vitro experiments show that these mechanical deformations can also lead to the generation of signals which increase osteoblast proliferation at certain stages of differentiation [81]. Cyclic tensile strain also regulates the expression of RANKL and OPG by osteoblast-like cells [82]. The proliferation and differentiation of osteoclasts in murine marrow cultures have also been shown to be inhibited by mechanical deformation [83]. 

Local production of parathyroid hormone-related peptide (PTHrP) by cells of the osteoblast lineage could also be an important local mediator of strain. Mice with deletion of the PTHrP gene in cells of the osteoblast lineage have osteopenia and decreased bone formation [84]. The expression of PTHrP is induced by stretch in osteoblasts [85]. PTHrP is expressed in the periosteum, particularly at the sites of tendon insertion [86]. We observed that PTHrP expression was decreased in the femur and tibia periosteum after 3 days of tail suspension in the rat [87]. As PTHrP has actions similar to PTH on osteoblasts and osteoblasts precursors, cyclical local PTHrP production with periodic mechanical stimuli could be one of the stimulators of bone formation at the periosteal level in response to loading. Because of the cooperation of strain and PTH receptor in cell signaling, a periodic increased PTHrP secretion by strain could amplify the response to strain itself.

## 5. Disuse

Reduced weight bearing in a microgravity environment induces bone loss in weight bearing bones [88]. Vico et al. [89] reported BMD results obtained by pQCT at the distal radius and tibial sites in 11 cosmonauts who completed a 6-month space mission. No significant changes were observed in the radius, while there was a variable but significant decrease in the tibia both for cortical (−0.4 to −4.3%) and trabecular (−0.4 to −24.0%) bone. Recovery was only incomplete 6 months after flight. Bone loss during flight results from uncoupling of bone remodeling, as shown by biomarkers [90, 91]. Mature rats of both sexes submitted to simulated weightlessness (hind limb suspension) also have a decreased periosteal apposition and trabecular bone formation, with increased bone resorption [92]. A decreased bone formation in microgravity could result from a shift of pluripotential mesenchymal stem cells differentiation from osteoblastogenesis to adipogenesis [93]. 

Prolonged bed rest leads to losses approaching those observed in microgravity, for instance 1.2% in the pelvis and 0.4%/month in the legs in the study of Leblanc et al. [94], and 1.2%/month for the trochanter in the study of Zerwekh et al. [95]. Zerwekh also observed an increase of resorption surfaces and a decreased bone formation at bone histomorphometry, and a significant increase of biological markers of resorption. Interestingly, there was also a significant increase of BMD in the head. This gradient in BMD changes probably results from fluid shifts in supine position or in microgravity [96]. Recovery was only partial after six months of remobilization [94]. 

**Figure 2:**
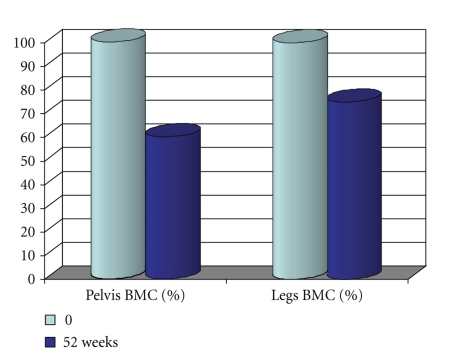
Disuse produces a dramatic bone loss in paraplegic patients, more so in trabecular rich bone areas (pelvis) than in areas containing relatively more cortical bone (femurs and tibias) (redrawn from Wilmet et al. [97]).

For those remaining on earth, the most dramatic and constant bone loss is that observed in the lower part of the body after a spinal cord section (0.5–1%/week in sub-lesional areas rich in trabecular bone, [97, 98]) (Figure 2). This loss is greater than that observed during simple immobilization, so that it could be caused in part by a modification of the neural control of bone mass which superimposes on disuse [99]. The loss is exponential and bone mass tends to stabilize after 1 year for trabecular bone, after 2-3 years for cortical bone [100]. Passive mechanical loading (assisted standing) seems to partially preserve bone mass in the paralyzed areas, particularly in the femur shaft [101]. In one study performed in patients with a spinal cord section 0.1 to 29.5 years duration, a treatment with alendronate 10 mg daily stabilized metaphyseal tibia BMD [102]. There was no increase of BMD in the paralyzed area, although an increase was observed in the spine. In a blinded placebo controlled study in acute spinal cord section, alendronate 70 mg/week mitigated bone loss in the sublesional areas, but the inhibition of bone loss was only partial [103]. We also observed only a partial response to weekly alendronate in an observational study in acute paraplegic patients [104]. 

The loss associated with hemiplegia is more important in the upper limbs. In a longitudinal study of 32 patients, followedup for a mean of 3 months, there was a 12% decrease of BMD in the paretic arm and a 5% decrease of BMD in the paretic leg [105]. The nonparetic arm lost 3.5% and the nonparetic leg 2%. Similar results have also been reported by Sato et al. [106]. These patients have an increased risk of hip fracture [107], due to bone loss, locomotor problems, and vitamin D insufficiency. 

A more frequent situation is that therapeutic bed rest is associated with bone loss [108, 109]. In Heaney's retrospective analysis of trials with risedronate, the annualized loss in patients who had to be hospitalized for a severe event was maximal at the trochanter, −2.7%/year versus −0.7%/year in nonhospitalized placebo controls [109]. Bone loss in osteopenic bedridden older patients probably contributes to the weakening of their skeleton. In Heaney's study, the loss was abolished in risedronate-treated patients.

## 6. Exercise and Bone Health

So, the deleterious effect of disuse is evident. Conversely, though experimental work shows that loading bones triggers bone formation and induces bone apposition, demonstrating a positive effect of exercising on the skeleton is much less evident, particularly in adults. Long-term exercise is associated with a higher skeletal mass in the young [110] and in the older [111]. However, as underlined by Forwood “exercise is not synonymous with mechanical loading” [112]. “Exercise” covers a large spectrum of varied activities, from wandering to highly demanding competition, and the level or the kind of exercise which best simulates specific loads applied directly to bone in the laboratory is not precisely known; thus, a great variability of effect is expected according to the type of exercise performed. For instance, even among athletes, while runners had an increased whole body and legs BMD than the general population, road cyclers had a lower bone density of the spine [113, 114] and hip BMD decreases in competition male cyclists followed for 1 year [115]; the expected effect is site specific; there are systemic effects which can interfere with the simple action of loading: alteration of calcium metabolism, with increased PTH secretion and an increased serum phosphate concentration [116, 117]; energy balance [118]; endocrine problems in super athletes, who have a decreased hypophyseal function [119]. On the other hand, exercise programs giving to bones the kind of signal which has been proven to stimulate bone formation (sufficient load applied at a sufficient rate and frequency) would be difficult to follow by the majority of the population, and particularly the elderly, most concerned by osteoporosis and fractures. Most of the evidence about exercising and fractures comes from epidemiological case finding studies and nonrandomized, thus biased, longitudinal series. The few randomized prospective longitudinal studies are by essence not blinded, of short duration and most often not powered to study the effect on fractures. The subject has been reviewed by Karlsson in two papers summarizing the available evidence for an antifracture efficacy of exercise [120, 121].

### 6.1. Exercise during Growth

Several studies have shown a higher BMD in athletes compared to the general population. In male weight lifters, bone mineral density is increased in the arms and legs, while it tended to be lower in the skull [122]. Female gymnasts have an increased bone mineral density in the arms, legs, and spine, and the increase is related with the length of training [123]. Soccer players were found to have a higher femoral bone mineral density [124]. 

As these population studies are subject to different bias, the most convincing effect of exercise on bone resistance come from studies of a site-specific effect. Life long tennis players of both sexes have a greater cortical thickness of the dominant versus contralateral arm [125, 126]. The effect on BMC is related to bone enlargement, and not to an increased volume bone mineral density. The differences are greater if exercising has started before puberty [127], and the enlargement of the periosteal envelope is greater in males than in females [128], an observation which concords with experimental data showing an inhibitory effect of estrogens on periosteal apposition [55, 56]. The increase in bone mass is associated with that of muscle mass [129]. The same conclusions were reached by Ducher et al., but in their study, tennis playing had also a positive effect on bone mineral density of the distal dominant radius, in children and adults [130]. 

Independently of high level sport training, several cross-sectional and observational longitudinal studies establish a relation between the level of physical activity and bone mass and geometry. An observational longitudinal study in boys and girls aged 5 to 11 years showed that the duration spent per day in moderate to vigorous physical activity, registered using an accelerometer, is an independent determinant of femur neck cross-sectional area measured by DXA and of the section modulus Z, an index of bending strength calculated from DXA measurements, even after taking into account lean mass, at least in boys [131]. Tobias has also observed that bone geometry was related to physical activity in a cross-sectional study of 4457 11-year-old children [132]. An observational longitudinal study conducted in 154 adolescent subjects (8 to 15 years of age at entry) at the University of Saskatchewan has shown a positive relationship between the estimated amount of physical activity during adolescence and total body, lumbar spine, and total hip BMC measured at the end of the adolescence growth spurt. The gain (8 to 10%) was maintained in young adulthood, perhaps because the physical activity profile remained unchanged across the groups [133]. At the end of growth, the GOOD study has shown that, in young adult men (18.9 year old), there was a positive relation between the amount of physical activity, cortical bone size, a BMD of the spine, femoral neck, radius and total body, and trabecular but not cortical vBMD [134]. Threshold amount of physical activity was 4 h/week, and the effect was larger if training was started before 13. 

**Figure 3:**
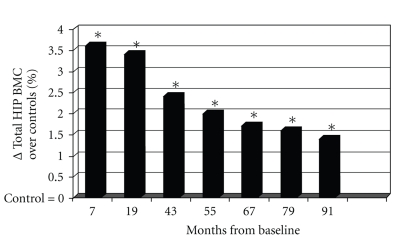
BMC increases more in children submitted for 7 months to a program of impact exercise (jumps) than in their control peers who had nonimpact activities. Age at onset was 8 years. Seven years after the end of the program, a small benefit of the impact activities was still detectable. Reproduced from Gunter et al. [142], with kind permission of Wiley.

Longitudinal controlled studies lead to more mitigated results. Recently, a study comparing 53 girls aged 7–9 years who volunteered for a 1 year exercise intervention program of 200 minutes per week did not have an increased BMD or better geometric hip parameters than their controls [135], contradicting previous results from the same group studying the lumbar spine [136] and from other groups [137–139]. A school-based 16-month randomized, controlled physical activity intervention program was also found to increase the distal tibia bone strength in prepubertal boys, but not in girls [140]. The most evident positive effect of physical exercise was observed in prepubertal children submitted to high impact exercise (jumping), even for only a few months [141]. The mean gain was 3.5% higher in the jumpers than in the controls at the end of the program. These children were then followed annually. Though there was a rapid loss of the gain during the 3 months following the end of the program, a small gain persisted after 7 years in the exercised group [142] (Figure 3). 

The heterogeneity of the results could result from many confounding factors, such as compliance, the sites studied, the type of exercise, age and sexual maturity at the beginning of the program, length of intervention, the basal level of physical activity, or the variation of lean and fat mass. 

Several nutritional factors might also interact with physical activity to change bone acquisition. One of these is calcium nutrition [143, 144]. However, in a randomized controlled study of 1-year duration, a calcium supplement of 500 mg per day did not change mineral volumetric BMD increases in 10-year-old female gymnasts [145]. Another important factor is protein nutrition. A recent epidemiological study by Chevalley et al. [146] showed a strong interaction between protein intake and physical activity in prepubertal children: boys having both a protein intake and a level of physical activity above the median had the highest BMD at several skeletal sites. In this study, no interaction was found with calcium intake, probably because all participants had a calcium intake above the desirable level.

### 6.2. Adult Life

#### 6.2.1. Maintenance of Bone Gain Acquired during Growth and Effect on Life-Long Fracture Risk

Theoretically, attaining a higher peak bone mass can protect against osteoporosis and fractures later in life. This is true only if the beneficial effect of high level physical activity in childhood is maintained throughout adult life. Animal studies show that it is the case only if “moderate” activity is continued [147–149]. heterogeneity can result from differences in training program [150]. The 5-year followup study of racket sports players has shown that some beneficial effect on bone mass can persist [151]. Former gymnasts retained an advantage in terms of BMD up to 12 years after retirement [152, 153]. Former male young athletes still had higher BMD 4 years after cessation of their career than controls [154]. Other reports are less optimistic. The sonographic parameters measured at the calcaneum decreased significantly in runners one year after stopping training [155]. Cross-sectional observations at longer time in soccer players show that the gains acquired during growth are lost after the termination of physical training, and that the fracture risk was not significantly lower after the age of 50 than in a control group without a history of high level sport activity [156]. 

The problem of how lifetime exercise influences BMD in women has been addressed in a study of mother-daughter pairs [157]. Lifetime exercise was estimated retrospectively from a questionnaire in 25 mother-daughter pairs and introduced in a multiple regression model as possible determinants of total, axial, and peripheral BMD. Weight-bearing exercise was significantly correlated with total and peripheral BMD in the daughters, but not in the mothers, suggesting that with age other variables become dominant as determinants of BMD, such as body weight, calcium nutrition, or estrogen use. However, in another cross-sectional study, Uusi-Rasi et al. using peripheral QCT observed that postmenopausal women (mean age 67) with a higher level of physical activity had increased vBMD and mechanical resistance of the tibia [158].

#### 6.2.2. Effect of Physical Activity during Young Adult Life on Bone Mass

A 10-year observational study by Bakker et al. [159] has shown that the evolution of lumbar BMD was related to ground reaction forces resulting from physical activity in young men, but not in women. The effect was small. In a randomized controlled trial on premenopausal middle-aged women (35–40 year old), high impact exercise (running, walking, jumping) practiced for 60 minutes three times a week for 1 year correlated significantly with BMD of the femur hip and trochanter when acceleration generated by impact was higher than 3.9 g, which is the case for fast running and jumping [160]. The exercised group also increased more bone circumference of the mid-femur. The changes in the trained group were minimal, but significantly related to the number of impacts [161].

#### 6.2.3. Prevention by Physical Activity and Exercise of Bone Loss Resulting from Menopause and Ageing

**Figure 4:**
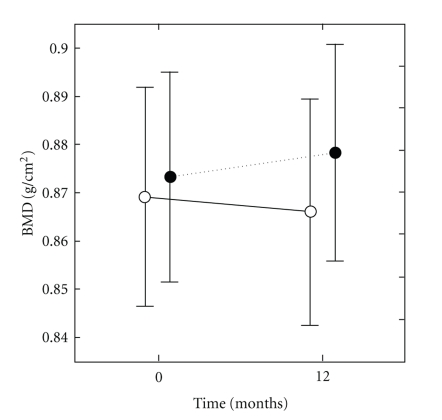
Evolution of hip BMD in a group of osteoporotic postmenopausal women randomized into a fast walking program (filled circles, *n* = 48), compared to 44 controls (open circles). The slight difference at one year was significant (*P* = .04). Reproduced from Bergström et al. Osteoporos Int 2008; 19: 177-83 [169], with kind permission of Springer.

Can exercise prevent menopausal bone loss and loss associated with ageing? A clue indicating that loading could prevent bone loss caused by hypogonadism is the increase of bone density at weight-bearing sites in amenorrheic athletes as opposed to a decrease at nonweight-bearing sites. In girls with anorexia nervosa, the loss of BMD is significant at all sites [162]. Also, exercise can prevent bone loss in ovariectomized rats [163]. However, a comparison of the effect of a standardized jumping exercise in pre- and postmenopausal women led to the conclusion, coherent with the physiological data, that the effect of exercise on BMD was blunted after menopause [164]. An unexpected finding in this study was that estrogen replacement for at least 12 months did not change the response in postmenopausal women. Thus, age rather than estrogen deficiency could be the cause of the decreased response, but the median age of postmenopausal women was only 55. A meta-analysis realized in 2000 and including 21 controlled randomized studies in postmenopausal women (median mean age 59) showed that training decreased slightly bone loss of pre- and postmenopausal women without HRT at the spine and femoral neck, both for impact and nonimpact exercises [165]. The more recent meta-analysis of Martyn-St and Carroll [166] based on 15 randomized controlled trials in postmenopausal women showed a significant but small positive effect (+0.006 g/cm^2^) at the spine, and no significant effect for the hip and femoral neck. In a recent prospective study [167], early postmenopausal women (52 years old) were randomly assigned to two groups, one with high demanding exercises (weight-lifting), and a control group. Each group had two arms according to the self-selected treatment with HRT. At the end of the study, of one-year duration, spine BMD was significantly more decreased (−3.6%) in the control group without HRT than in the three other groups. The exercising groups, with or without HRT, had a small bone gain at the spine (+0.7%) that was significantly different of the small loss (−0.7%) in the HRT group without training. There was no additive effect of training and HRT, as in previous studies performed in older women [168]. Recently, a randomized study in postmenopausal osteopenic and osteoporotic women 45 to 65 years old has shown a small but significant beneficial effect of fast walking (3 × 30 min/week) and physical training twice per week during 1 year on total hip BMD [169] (Figure 4). Another recent cross-sectional study of the relation between physical activity and bone density and strength has shown no association with areal BMD; the only positive association was between bone resistance and the amount of physical activity at the femoral diaphysis site [170].

#### 6.2.4. Is Physical Exercise Useful in the Treatment of Postmenopausal or Senile Osteoporosis?

There is evidence from animal experiences that the adaptation of bone to mechanical loading decreases with age [171]. This decreased sensitivity to loading could indeed be one of the physiological bases of senile osteoporosis. Thus the stimuli required to obtain an increase in bone resistance could be higher in the old than in the young. On the other hand, vigorous exercising in the seniors can lead to injuries [172]. The results of a 12 months randomized controlled trial testing resistance and balance-jumping training in 70 to 79 years old women without osteoporosis did not show any significant beneficial effect on BMD or bone geometry at the level of the femur neck or tibial shaft [173]. However, there was an improvement of physical performances in the trained groups which could contribute to fracture prevention. 

Physical exercise alone will probably never be a treatment of established osteoporosis, but it is important to clarify the interaction between loading and the pharmacological interventions in osteoporosis. A recent experimental study in ovariectomized rats showed that rats treated with alendronate and exercised for 14 weeks on treadmill had a significantly higher BMC normalized for body weight at L4 and at the proximal and mid femur than animals treated with alendronate alone. Exercised animals also had a higher femur cortical area and cortical thickness, by simultaneous reduction of the medullary canal (alendronate) and expansion of the periosteal perimeter (exercise) [174]. In a 1-year randomized trial on 164 early postmenopausal women submitted to a progressive jumping exercise plus or minus alendronate, exercise alone had no significant effect on bone mineral density. There was no significant additive effect of alendronate and training on bone mineral content at different skeletal sites, but exercise increased bone strength of the distal tibia [175]. An additive effect seems clearer in patients at risk of glucocorticoid-induced osteoporosis. While alendronate alone only stopped bone loss in transplanted patients at risk of glucocorticoid-induced osteoporosis, the addition of resistance exercise increased total body, lumbar spine, and femur neck bone mineral density in these patients [176, 177]. 

However, more than an additive effect of physical training with bisphosphonates, the main question is if there is a decreased efficacy of bisphosphonate treatment in patients with a decreased level of physical activity. An experimental work in dogs with a 12-month forelimb immobilization suggests that the increased resorption of disuse is not completely abolished by risedronate [178]. The trials using bisphosphonates in patients with a spinal cord section show that they do not completely inhibit bone loss when the lesion is recent [103] and that they stop bone loss but do not increase it in the paralyzed areas when the lesion is older [102]. 

Taking into account the interaction of PTH signaling with mechanical loading on bone cells [36–38, 179–181], there could be an interaction between physical activity and treatment of osteoporosis with PTH fragments, but this was not studied at present in the clinic. Experimental studies on rats are encouraging [182, 183]. Using a model of hind limb suspended rats, Turner et al. have shown that though PTH prevented trabecular bone loss [41], weight bearing was necessary for PTH to increase bone formation in cortical bone [42].

### 6.3. Simulating Mechanical Forces

Many attempts have been done to limit the loss of bone resulting from disuse, in disabled patients, and in astronauts who could suffer from a long period of weightlessness. These attempts have frequently been disappointing. Studies showing bone loss in astronauts have been done while they were subjected to an intensive exercise program both before and during flight [184]. Resistive exercise during bed rest only partially mitigated bone loss in the diaphysis and epiphysis of the tibia in volunteers subjected to 90-day bed rest [185]. Functional electrical stimulation cycling applied to patients with spinal cord injury can partially reverse bone loss in the distal femur [186].

 A special mention must be given to the recent attention which has been given to vibrations. The underlying idea is that much of the mechanical signaling acting to modify bone mass and trabecular architecture does not necessary come from extreme strains, but also from small strains resulting from constant muscle activity, such as that involved in maintaining posture [187]. Indeed, disuse osteoporosis induced in rats by tail suspension can be prevented by brief exposure to extremely low magnitude mechanical stimuli [16]. A preventive effect of vibrations was also observed on the increased endosteal resorption and decreased strength of the femur and tibia associated with ovariectomy in adult rats [188]. In 2002, Rubin et al. submitted for 20 min/day 5 days a week the hind limbs of sheep to ground vibrations with a peak-to-peak acceleration of 0.3 g and a frequency of 30 Hz. The strain generated in the tibia was of the order of 5 *με*, 500 times less than that induced by walking. The animals were studied by DXA at different time points; after one year, the femurs were analyzed ex vivo by DXA and pQCT, and submitted to histomorphometry. Both pQCT and static histomorphometry showed an increase of trabecular bone volume of 30%, which was highly significant. Bone formation and mineralizing surface were also significantly increased [189]. The program also had a positive effect on bone resistance [190]. A similar program was applied to 70 young postmenopausal women who were randomized to a vibrating platform with the same characteristics as the experimental one described above: the subjects who had the highest compliance gained 0.04% in femoral BMD and 0.1% in the lumbar spine BMD, as compared with a 2.1 and 1.6% BMD loss in the control group, with a greater benefit in the women with the lowest BMI [191]. Using a vibrating platform with higher ground reaction forces (peak acceleration 2.3 to 5.1 g) in healthy women 60–70 years old, Verschueren et al. observed a significant gain of hip BMB in the experimental group than in a group submitted to resistance training or a control group (0.93%, versus 0.14% in resistance training and a loss of 0.62% in controls) [192]. These results are at variance with a previous study which did not show a significant effect on BMD in a group of younger individuals for whom the duration of the stimulus was shorter (4 min, versus 20) [193]. 

Experimental data in mice have also shown a beneficial effect of brief application (15 min/day) of high frequency low-intensity vibrations on the growing skeleton: after 3 weeks, bone resorption was decreased, and after 6 weeks, bone mineralizing surface of the proximal metaphysic of the tibia was significantly greater, so as trabecular bone volume, periosteal, and cortical bone area, and the moment of inertia [194, 195]. A positive effect was also observed on muscle mass. A controlled trial conducted in 48 young women (mean age 17 years) with low BMD showed a borderline significant effect on muscle mass, spine cancellous volume BMD measured at CT, and femur cortical bone area [196]. At present, no study has examined the effect on fractures.

## 7. Summary and Conclusion

Mechanical loading is a major regulator of bone mass and geometry. Sensing and transduction of strains are essential to maintain bone health. The final result of loading is to decrease osteoclastic resorption and to increase formation at places where more strength is needed to resist loading. Thus, interventions on mechanical forces could be the most physiological way to increase the mechanical resistance of bone and prevent or treat osteoporosis. 

The main sensor of mechanical forces is the osteocytes network, which transmits orders to effector cells, osteoclasts, and osteoblasts, by the secretion of several cytokines which modulate the concentration of the bone microenvironment in OPG and RANK-L, and in IGF's. Key molecules in sensing and transducing are calcium channels, MAP kinases, particularly ERK 1/2 and the estrogen receptor *α*. The response of bone to mechanical solicitations will depend on the quality of these solicitations, both in terms of intensity and of frequency. Because of desensitization of the system with continuous loading, intermittent activity is expected and has been shown to be more efficient. The response will also depend on the sensitivity of the sensor, which decreases with age and is modulated by the hormonal environment, particularly estrogens. The maximal capacity of cells of the osteoblastic lineage to respond to the signals is also a determinant factor which could limit the efficacy of mechanical interventions in adults and seniors [184]. Thus, though a deleterious effect of disuse is evident at all ages, a beneficial effect of increased loading through exercising is much more difficult to establish. The clearest conclusion to be drawn from the studies on exercising and bone is the beneficial effect of exercise during growth on peak bone mass. Intensive training during growth favors higher bone mass and stronger bone geometry, particularly during the prepubertal period. It is still unclear if the benefit of intensive training in childhood is maintained after retirement, and for how long. Probably that a threshold level of exercise during adulthood is necessary to maintain the benefit in terms of bone mass and geometry, but the level of this threshold remains unknown at present. Thus, exercise programs in school are probably valuable in terms of osteoporosis prevention but only if campaigns are organized to push adults to maintain a sufficient level of physical activity throughout life. What is “sufficient” is not really well defined, but 30 minutes to 1 hour walking per day would probably help. Studies on exercise programs in adults have shown small, not always significant effect on BMD and sometimes on bone geometry. These programs are quite difficult to follow, compliance is usually low and it would be unrealistic to implement them systematically as part of osteoporosis treatment. As it is probable that both anti resorptive treatments and treatments stimulating formation are more efficient if bones are challenged by mechanical strains, patients receiving a treatment for established osteoporosis should be advised and encouraged to maintain a reasonable level of physical activity. Trials adding vibrations to classical pharmacological treatments of osteoporosis would be interesting. 

Besides the effect of physical activity on bone, it also affects muscle mass and force, an effect which will contribute to fall and fracture prevention. 

The mechanisms of sensing and transducing begin to be known in sufficient details to allow to think of pharmacological interventions which could simulate loading [197]. However, these mechanisms are common to many cells, and such interventions will have to be sufficiently targeted to bone cells not to interfere with other essential physiological processes.

## Figures and Tables

**Figure 1:**
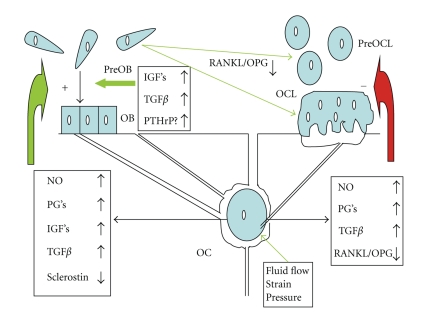
Mechanotransduction in bone. OC: osteocyte; OB: osteoblast; OCL: osteoclast; Pre-OB: preosteoblast; Pre-OCL: preosteoclast; PG's: prostaglandin (E2 and I); RANK-L: receptor activating NF*κ*B-Ligand; OPG: osteoprotegerin; IGF: insulin-like growth factor; TGF: transforming growth factor; Scl: sclerostin; PTHrP: parathyroid hormone related peptide. Osteocytes sense the fluid flow induced by loading in the lacunocanalicular system; this signal modulates the secretion in the bone microenvironment of factors which can increase bone remodeling while stimulating osteoblast differentiation and activity (green arrows) and decreasing osteoclast activity (red arrow), resulting locally in a positive bone balance.
